# Potential fibrinolytic activity of an endophytic *Lasiodiplodia pseudotheobromae* species

**DOI:** 10.1007/s13205-016-0428-4

**Published:** 2016-05-19

**Authors:** Vineet Meshram, Sanjai Saxena

**Affiliations:** Department of Biotechnology, Thapar University, Patiala, Punjab 147004 India

**Keywords:** *Aegle marmelos*, *Botryosphaeria* sp., Clot busters, Endophytic fungi, ITS–rDNA

## Abstract

Cost-effective, fibrinolytic agents possessing least or no antigenic properties are much in demand for their prospective use as clot busters in thrombolytic therapy. The present communication explores the potential of 22 endophytic *Botryosphaeria* species isolated from stem of *Aegle marmelos* for their fibrinolytic potential. Only nine fungal isolates exhibited proteolytic activity out of which only four possessed plasminogen-independent fibrinolytic activity. The endophytic fungal isolate #1088 AMSTITYEL showed maximum in vitro proteolytic and fibrinolytic activity with a halo formation of 153.86 and 113.04 mm^2^, respectively. The partially purified protein exhibited a fibrinolytic activity equivalent to 6.51 U/ml of plasmin. SDS–PAGE of the dialysed fraction of #1088 AMSTITYEL resolved into six bands ranging from 26 to 80 kDa. Fibrin zymogram exhibited that a single band of molecular size ~80 KDa possessing the fibrinolytic activity. Furthermore, the bioactive isolate #1088 AMSTITYEL was identified as *Lasiodiplodia pseudotheobromae* using classical and molecular taxonomic tools.

**Electronic supplementary material:**

The online version of this article (doi:10.1007/s13205-016-0428-4) contains supplementary material, which is available to authorized users.

## Introduction

Blood clots are formed as a preventive response of the human body to avoid excessive bleeding at the site of injuries and wounds (Raafat et al. 2012). Fibrin is the major protein involved in blood clot formation, which is formed from its precursor fibrinogen by the enzymatic action of thrombin (EC 3.2.21.5) and is further lysed by plasmin (EC 3.4.21.7) (Kim et al. 1996). Under normal condition, blood clot formation and its dissolution are meticulously regulated by the biological system; however, impairment in this doctrine mechanism leads to thrombosis where the insoluble blood clots do not get hydrolysed and adhere to the wall of the blood vessels ultimately resulting in cardiovascular disorders (CVDs), such as myocardial infarction, high blood pressure, ischemic heart disease, stroke, and so on (Simkhada et al. 2010). These CVDs toll over 18 million lives annually and are expected to rise to 23.6 million by 2030 (Mander et al. 2011). The current armamentarium of drugs involved in thrombolytic therapy involves intravenous administration of streptokinase and urokinase, which instantly opens up the blood flow caused due to fibrin blockage, thus reducing the chances of morbidity and mortality (Raafat et al. 2012; Kim et al. 1996). Based on their mechanism of action, thrombolytic drugs are divided in two types: the former is plasminogen activators and urokinase, which converts dormant plasminogen into active plasmin that hydrolyses the fibrin clot and the latter one is plasmin-like proteins that directly hydrolyse the blood clots (Simkhada et al. 2010). Despite their widespread use, these drugs suffer certain shortcomings, such as low specificity to fibrin, internal bleeding, short half-life and cost effectiveness (Wu et al. 2009b). Therefore, there has been a continual search for safer and cost-effective fibrinolytic agent from diverse sources. To date, fibrinolytic agents have been isolated and characterised from different sources, including plants, animals, microbes and fermented foods (Deng et al. 2010; Choi et al. 2011). Microbes are considers as a lucrative source of fibrinolytic agents due to their diverse biochemical nature, feasibility of mass culture and ease in genetic manipulation. Hence, over the last two decades, fibrinolytic enzymes have been successfully discovered from microbial sources, such as bacteria, actinomycetes and fungi (Peng et al. 2005; Wu et al. 2009b; Mander et al. 2011).

Endophytic fungi are special group of microorganisms residing within the plants, which play an important role in defending the host plant against stresses, such as pathogenic invasions and drought. The genetic recombination of the endophytes with the host plant enables them to mimic the biological properties of their host and produce analogous bioactive metabolites. Production of an anticancer compound ‘taxol’ in the culture broth of *Taxomyces andrenae* isolated from *Taxus baccata* is the best example of it (Daisy and Strobel 2003; Zhang et al. 2006). Exploration of enzymatic properties of these endophytic fungi is a novel and largely under-explored aspect, and there exist scanty literature on screening and isolation of novel fibrinolytic enzymes from these microorganisms. Endophytic fungi, such as *Verticillum* sp*., Fusarium* sp. and *Bionectria* sp., are amongst few reported to possess in vitro fibrinolytic activity (Li et al. 2007; Wu et al. 2009a; Rovati et al. 2010).

In this study, we undertook an in vitro screening program to assess the fibrinolytic potential of 22 endophytic fungal isolates of *Botryosphaeria* species isolated from the Western Ghats in India. This study is also the very first report of an endophytic *Botryrosphaeria* species exhibiting fibrinolytic activity. Fungal fibrinolytic enzymes may be exploited as a substitute to streptokinase, which is of bacterial origin and currently being exploited as a thrombolytic agent by the pharmaceutical Industry.

## Materials and methods

### Plant sample collection and isolation of the endophytic fungi

Healthy twigs of medicinal plant *Aegle marmelos* (Bael, golden apple) belonging to family *Rutaceae* were collected from the conserved rain forest area of Western Ghats, geographically located between 11°59′ and 11°99′N, 77°8′ and 77°14′E (Yelandur, BRT wildlife sanctuary, Karnataka) and 11°35′–11°51′N, 76°14 02′–76°27′E (Muthanga Wildlife Sanctuary, Wayanad, Kerala) during rainy season of 2009. The endophytes from the twigs were isolated by previously described method (Schulz et al. 1993). Briefly, the twigs (5 cm) were surface sterilized with sequential washing with 2 % sodium hypochlorite for 3–5 min followed by 70 % ethanol for 2 min and then with 30 % ethanol for 1 min. The surface sterilized samples were then cut into 1-to-2-mm segments with the help of sterile blade and placed over water agar plates under aseptic conditions. These were then incubated at 26 °C for 10 days. The efficiency of surface sterilization was confirmed by imprinting the surface sterilised plant part over the PDA plate. The absence of fungal growth on the respective medium portion confirmed the efficacy of surface sterilization. Maximum of eight segments was inoculated each plate. The fungus emerging out of the host tissue was aseptically subcultured to fresh PDA plate, so as to obtain pure isolates. These pure isolates were further preserved on PDA slants supplemented with 10 % glycerol (Naik et al. 2008; Zhang et al. 2010). The colonisation frequency (% CF) of endophytic fungi was calculated using the formula % CF = (*N*_col_/*N*_t_) × 100, where, *N*_col_ = number of segments colonised by each fungus and *N*_t_ = total number of segments inoculated (Gond et al. 2007). Isolation was performed in duplicate for samples collected from each location. The endophytic fungal isolates were encoded based on the host plant, its part and the place from where it was collected, e.g. #6 AMSTYEL, where #6 refers to segment number; AM stands for *Aegle marmelos*; ST refers to stem, YEL signifies to Yelandur, Karnataka (sample collection site). Furthermore, #6 AMSTYEL belong to the first phase of isolation, whereas #1088 AMSTITYEL denotes to the second phase of isolation (10 refers to the second set of isolation, whereas 88 refers to the segment number). Similarly, #20 AMSTWLS refers to 20th segment from stem tissue of *Aegle marmelos* collected from Wayanad, Kerala.

### Preparation of cell-free culture filtrates

Liquid cultures of 22 endophytic fungal isolates were produced to assess the extracellular in vitro fibrinolytic activity. Briefly, the method comprised of an aseptic inoculation of 50-ml presterilised Czapek Dox Broth (HiMedia, India) with 5-mm mycelial disc of 7-day old culture of the endophytic fungus followed by incubation at 26 ± 1 °C, 130 rpm for 7 days. After completion of the incubation period, the mycelial mass was separated using Whatmann filter paper, followed by centrifugation at 8000 rpm (Hitachi CF 15 RX II series, Japan) for 10 min at room temperature. The supernatant was then passed through 0.22-µm nitrocellulose membrane (GE Healthcare Life Sciences, USA). These cell-free culture filtrates were further tested for their in vitro proteolytic and fibrinolytic potential by plate assays (Ali and Ibrahim 2008).

### In vitro proteolytic and fibrinolytic assays

The cell-free culture filtrates were tested for their proteolytic and fibrinolytic activity by plate assays (Ali and Ibrahim 2008). The production of a proteolytic and fibrinolytic enzyme was indicated by formation of a halo around the well where the test sample was dispensed.

### In vitro proteolytic activity

Briefly, in vitro proteolytic activity was performed on agar plates comprising of 1 % skimmed milk (HiMedia, India) dissolved in 50 mM Tris–HCl buffer as substrate together with 1 % agar (w/v). After solidification, 5-mm wells were scooped, and 30 µl of cell-free culture filtrate was dispensed on agar plate and incubated at 37 °C for 18–24 h. No culture filtrate was added in the control well. After incubation period, proteolytic activity was evaluated by measuring halo diameters. For screening purposes, proteolytic activity was expressed as the clear zone area (in mm^2^) (Rovati et al. 2010).

### In vitro fibrinolytic activity

Fibrinolytic activity was determined following a modified procedure described by Astrup and Mullertz on plasminogen rich and plasminogen free plates (Astrup and Mullertz 1952). Briefly, plasminogen free plates were prepared by mixing 5 ml of 0.5 % w/v fibrinogen (Calbiochem, Darmstadt, Germany) in 50-mM Tris–HCl buffer (pH 7.8) with 1 % agarose and 100 µl of thrombin (100 NIH U/ml, Sigma Aldrich, USA). Plates were allowed to stand for 30 min at room temperature to form fibrin clot. Plasminogen rich plates were supplemented with 5 U of plasminogen (Sigma Aldrich, USA). Thereafter, 30 µl of the cell-free extract was dispensed into 5-mm wells. Plates were incubated at 37 °C for 24 h. The control wells were devoid of culture filtrate. Clear transparent zone suggested fibrin degradation, and the potency of fibrin degradation is proportional to its diameter. After measuring the dimension of the clear zone, the number of units was determined according against a calibration curve of plasmin (from human plasma, Calbiochem, Darmstadt, Germany, Supplementary material 1). Average diameter was computed by diagonally measuring the clear zone around the well (Rovati et al. 2010; Choi et al. 2013). All the tests were performed in triplicate, and their mean and SD was calculated.

### Partial purification and characterisation

Based on the screening results, the endophytic isolate exhibiting highest fibrinolytic activity was selected for further studies. Solid ammonium sulphate (HiMedia, India) was slowly added to the culture broth (#1088 AMSTITYEL) to achieve 70 % saturation. The mixture was then incubated overnight at 4 °C, and the next day protein precipitate was collected by centrifugation at 13,000 rpm (Hitachi CF 15 RX II series, Japan) for 15 min at 4 °C. The precipitate was dissolved in minimum volume of 50-mM Tris–HCl buffer (pH 7.8). The crude enzyme was further dialysed against the same buffer overnight, and the dialysed fraction was recovered after centrifugation at 13,000 rpm for 15 min at 4 °C (Li et al. 2011). The fractions obtained after each step was tested for fibrinolytic activity via fibrin plate assay as described previously, while the protein content was determined by Bradford’s method using bovine serum albumin (HiMedia, India) as standard and measuring the absorbance at 595 nm (Bradford 1976).

The crude enzyme extract was subjected to sodium dodecyl sulphate polyacrylamide gel electrophoresis (SDS–PAGE) (Laemmli 1970). The molecular mass of the denatured enzyme was estimated using a standard broad range protein weight marker (Merck, Millipore, USA). The resolved proteins were detected in the gel by modified silver staining method (Blum et al. 1987). Fibrin zymography was performed according to the method of Kim et al. (1998). Resolving gel solution (12 %) contained 0.12 % (w/v) fibrinogen prepared in a total volume of 10 ml and centrifuged to remove insoluble impurities which were induced when SDS stock solution was mixed. Thrombin solution (1 U/ml) and TEMED (*N*,* N,* *N*′,* N*′-tetramethylethylenediamine) were added to the gel solution in final concentrations of 0.1 U/ml and 0.028 % (v/v), respectively. The enzyme was electrophoresed on a fibrin gel. The gel was then washed in 2.5 % Triton X-100 solution, incubated in zymogram reaction buffer [30 mM Tris (pH 7.4)] containing 200-mM NaCl, 10-mM CaCl_2_, and 0.02 % NaN_3_ at 37 °C overnight, stained with Coomassie Brilliant Blue R-250 (SRL, India) and, subsequently, destained. The cleared white area against the blue gel represents fibrin degradation.

### Taxonomic studies

Taxonomic identification of the selected endophytic fungal isolate exhibiting highest fibrinolytic activity was done using morphotaxonomic and molecular taxonomy.

### Molecular taxonomy

For genomic DNA extraction, about 0.1–0.2 g of cultured mycelia was scrapped off from 3-to-4-day old culture with sterile inoculation loop and crushed to very fine powder in pestle and mortar using liquid nitrogen. Furthermore, DNA extraction was done by using Wizard^®^ Genomic DNA purification kit (Promega,USA) following manufacturer instructions. Furthermore, the internal transcribed spacer (ITS) region 1, 5.8S, 2 was amplified using ITS 1 and ITS 4 primers (White et al. 1990). A 25-µl reaction mixture consists of 1 µl of extracted genomic DNA, 10 µM of each primer, 2.5 mM of dNTP, 1.5 U of *Taq* DNA polymerase in 10 X Taq buffer containing 25-mM MgCl_2_ (GeNei, Bangalore, India). The thermal cycling conditions consisted of initial denaturation at 96 °C for 5 min followed by 39 cycles of 95 °C for 45 s, 60 °C for 45 s, 72 °C for 45 s followed by final extension at 72 °C for 5 min (White et al. 1990). The ITS amplicons were examined using a 1.5 % agarose gel under UV light in Bio-Rad Gel documentation system using Quantity-1-D analysis software. The PCR products were purified with Wizard^®^ SV gel and PCR clean up system (Promega, USA) following manufacturer’s protocol. PCR products (500–600 bp) were sequenced from Chromus Biotech Labs (Bangalore, India). The final sequence was obtained by assembling the chromatograms using Sequencher ver. 5 (www.genecodes.com). The final sequence was submitted in GenBank under the accession number KJ 756377.

Sequence similarity search of ITS sequences of #1088 AMSTITYEL was performed using BLAST algorithm software (parameters for BLAST search: searched database: others (Nucleotide collection), BLAST algorithm–somewhat similar sequences (blastn), filter used—low complexity regions) at NCBI website. The ITS sequences were aligned with retrieved sequences of reference taxa obtained from BLAST using Clustal W option in MEGA 5. The alignment file involved 11 sequences, which comprised of 1 sequence under study, 9 sequences from BLAST search, which are representative sequences of respective genera/species and one *Mycosphaerella africana* species which served as an outgroup to root the tree. The aligned sequences were trimmed, so as to make alignment uniform, and the aligned files were then saved in FASTA and MEGA format. The evolutionary history was inferred using the maximum likelihood method based on BIONJ method with MCL distance matrix in MEGA5. A discrete gamma distribution was used to model evolutionary rate differences among sites [five categories (+*G*, parameter = 0.6046)]. All positions containing gaps and missing data were eliminated (Nei and Kumar 2000; Tamura et al. 2011).

### Morphotaxonomy

Morphotaxonomy of the endophytic isolate #1088 AMSTITYEL was done by growing it over PLA (Pine Leaf Agar) and incubated at 26 °C for 3–4 weeks in dark. The morphological and microscopic characters were critically observed and recorded. The microscopic characters were studied using a Nikon Eclipse 50i microscope (Nikon, Japan) coupled with CCD camera and measurements carried out using NIS elements D3.2 software. At least 30 observations were made per structure (Abdollahzadeh et al. 2010).

## Results and discussion

### Isolation of endophytic fungi

*Aegle marmelos* is an important medicinal plant as documented in ayurvedic pharmacopeia for variety of therapeutic properties, such as anti-inflammatory, anti-diabetic, hepatoprotective, and so on. It has recently been documented to possess anti-thrombotic activity too (Chougule et al. 2014). Based on the hypothesis that endophytes produce putative phytochemicals similar to their host, we chose to screen and evaluate the fibrinolytic activity of endophytic fungi from *A. marmelos*. In this study, 48 endophytic fungal isolates were isolated from the stem tissue of *A. marmelos.* They belong to different genera, including *Alternaria* sp., *Aureobasidium* sp., *Botryosphaeria* sp., *Fusarium* sp., *Pestalotiopsis* sp., and *Pheoacremonium* sp., and certain endophytic fungi, such as *Neofusicoccum parvum* and *Togninia* sp., were isolated in their teleomorphic form. *Botryosphaeria* (CF: 27.5 %) was the most dominant genus followed by *Fusarium* (CF: 21.25 %), *Pestalotiopsis* sp. (CF: 5 %) and *Pheoacremonium* sp. (CF: 3.75 %). *Botryosphaeria* sp. and *Pestalotiopsis* sp. were isolated from both the sampling sites, whereas *Fusarium* and *Aureobasidium* sp. were isolated only from Yelandur. Similarly, *Pheoacremonium* sp. and *Alternaria* sp. were only isolated from Wayanad wildlife sanctuary. This study was aimed at exploring the fibrinolytic activities of endophytic fungi particularly *Botryosphaeria* species isolated from *A. marmelos*. Several endophytes, such as *Fusarium* sp., *Alternaria* sp., *Rhizoctonia* sp., and *Curvularia* sp., have been reported from *A. marmelos. Fusarium* sp. was the most dominant endophytic fungal isolate followed by *Alternaria* sp. (Gond et al. 2007; Meshram et al. 2012). However, this is the first study of the presence and diversity of *Botryosphaeria* sp. as endophyte in *A. marmelos.*

### Screening for proteolytic and fibrinolytic activities

**Fig. 1:**
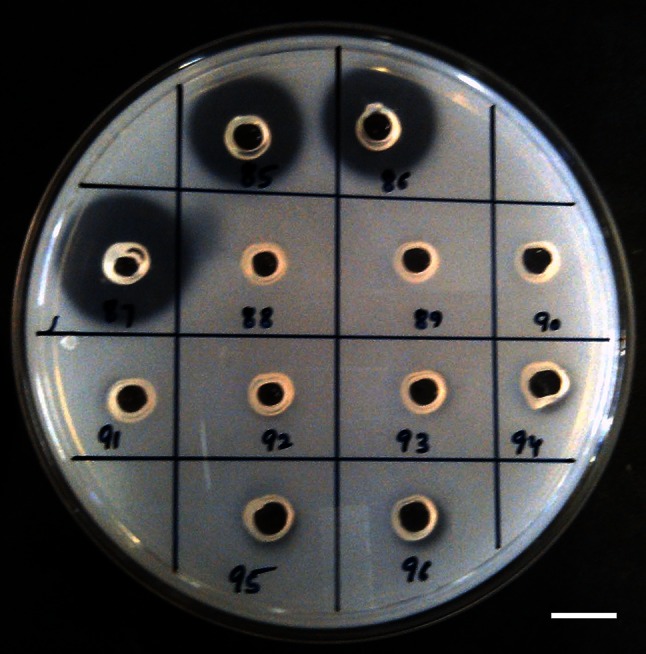
Screening for proteolytic activity (on 1 % w/v skimmed-milk agar plates) in culture filtrate of endophytic fungal isolates of *A. marmelos*. Well id: 85–87: #1088 AMSTITYEL; 88–90: #1082 AMSTITYEL, 91–93: #1079 AMSTITYEL; 94–96: control

**Fig. 2:**
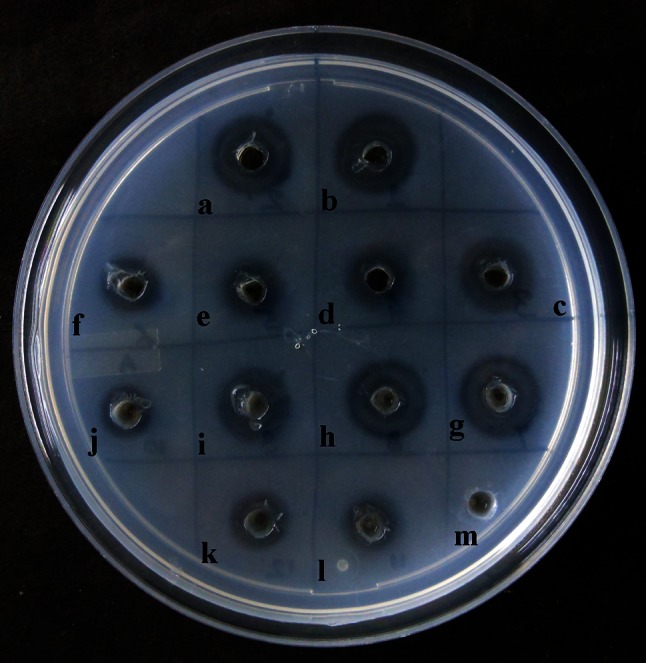
Screening culture filtrates of endophytic fungal isolates of *A. marmelos* for fibrinolytic activity. Well id:* a*–*c* #1095 AMSTITWLS;* d*–*f* #1013 AMSTITYEL,* g*–*i* #1048 AMSTITYEL;* j*–*l* #1088 AMSTITYEL, m: control

As evident from the screening procedure, cell-free culture filtrate of 40 % of the endophytic fungi exhibited proteolytic activity, while only 18 % of them exhibited fibrinolytic activity. Proteolytic and fibrinolytic activity of the cell-free culture filtrate was evident by formation of a clear zone or halo around the well there by depicting the degradation of respective substrate. Maximum proteolytic activity was shown by culture filtrate of fungal isolate #1088 AMSTITYEL followed by #11 AMSTYEL and # 1095 AMSTWLS (Fig. 1). The cell-free culture filtrate of only four endophytic fungi exhibited in vitro extracellular fibrinolytic activity. The maximum fibrinolytic activity was expressed by isolate #1088 AMSTITYEL with a fibrinolytic halo of 113.04 mm^2^. The fibrinolytic halo formation in the in vitro plate assay ranged between 50.24 and 113.04 mm^2^ (Table 1; Fig. 2). The cell-free culture filtrate exerted direct as well as indirect hydrolysis of fibrin clot. The zone of clearance was similar in both plasminogen rich and plasminogen free plates indicating that the enzyme activity does not work as tissue plasminogen activator (t-PA). Fungal fibrinolytic enzymes, such as CMase, Fu–P and verticase, are not a plasminogen activator, whereas herinase is a bifunctional enzyme (Li et al. 2007; Cui et al. 2008; Wu et al. 2009b; Choi et al. 2013). The fungal isolate #1088 AMSTITYEL exhibited a significantly better fibrinolytic profile when compared with *Bionectria* sp. (66.7 mm^2^) and *Cladosporium* sp. (58.3 mm^2^) (Rovati et al. 2010). Li et al. (2007) screened 23 endophytic fungi residing in *Trachelospermum jasminoides* for their fibrinolytic property. *Verticillium* sp. Tj33 was the most potential producer of fibrinolytic enzyme amongst the screened endophytes. Similarly, among 1075 screened endophytic fungi, fermentation extract of *Fusarium* sp. CPCC 480097 exhibited the strongest fibrinolytic activity (Wu et al. 2009b).

**Table 1:** Proteolytic and fibrinolytic activity of endophytic fungal species isolated from *A. marmelos*

Culture Code	Taxonomic identification	Place of sample collection	Area of halo formation (mm^2^)		Plasmin equivalence (U/ml)
			Proteolytic activity	Fibrinolytic activity	
#6 AMSTYEL	Lasiodiplodia theobromae*	Yelandur, Karnataka	–	–	–
#11 AMSTYEL	Neofusicoccum parvum*	Yelandur, Karnataka	113.04	–	–
#18 AMSTYEL	*Neofusicoccum* sp.*	Yelandur, Karnataka	63.58	–	–
#23(b) AMSTYEL	*Lasiodiplodia *sp.	Yelandur, Karnataka	–	–	–
#32 AMSTYEL	*Botryosphaeria* sp.	Yelandur, Karnataka	–	–	–
#1003 AMSTITYEL	*Botryosphaeria* sp.*	Yelandur, Karnataka	44.15	–	–
# 1013 AMSTITYEL	*Botryosphaeria* sp.*	Yelandur, Karnataka	63.58	63.58	1.41
#1032 AMSTITYEL	*Botryosphaeria* sp.	Yelandur, Karnataka	–	–	–
#1048 AMSTITYEL	Lasiodiplodia pseudotheobromae*	Yelandur, Karnataka	78.5	50.24	0.94
#1079 AMSTITYEL	Lasiodiplodia theobromae*	Yelandur, Karnataka	–	–	–
#1082 AMSTITYEL	Lasiodiplodia pseudotheobromae*	Yelandur, Karnataka	–	–	–
#1088 AMSTITYEL	Lasiodiplodia pseudotheobromae*	Yelandur, Karnataka	153.86	113.04	3.12
#20 AMSTWLS	*Botryosphaeria* sp.	Wayanad, Kerala		–	–
#25 AMSTWLS	*Botryosphaeria* sp.	Wayanad, Kerala	–	–	
#53 AMSTWLS	*Botryosphaeria* sp.	Wayanad, Kerala	–	–	–
#59 AMSTWLS	Botryosphaeria stevensii*	Wayanad, Kerala	94.98	–	–
#1095 AMSTITWLS	Lasiodiplodia theobromae*	Wayanad, Kerala	113.04	94.98	2.49
#1099 AMSTITWLS	*Botryosphaeria* sp.	Wayanad, Kerala	–	–	–
#1103 AMSTITWLS	*Sphaeropsis sapinea*	Wayanad, Kerala	–	–	–
#1104 AMSTITWLS	Barriopsis iraniana*	Wayanad, Kerala	38.46	–	–
#1111 AMSTITWLS	Barriopsis iraniana*	Wayanad, Kerala	–	–	–
#1118 AMSTITWLS	Barriopsis iraniana*	Wayanad, Kerala	–	–	–

^**#**^Standard errors were less than 5 % from three individual tests

* Identified using molecular taxonomy

### Partial purification and characterisation of fibrinolytic enzyme

Ammonium sulphate precipitation followed by dialysis, SDS–PAGE and fibrin zymography was used to assess the fibrinolytic potential of the extracellular proteins expressed by the endophytic fungal isolate #1088 AMSTITYEL. The amount of protein present in cell-free culture filtrate was 1804.17 mg. The specific fibrinolytic activity exhibited was 1.77 U/mg. The crude protein obtained after ammonium precipitation was 18.54 mg exhibiting 2.79 U/mg of specific fibrinolytic activity. Subsequent dialysis of crude protein leads to further purification of the protein fraction. Protein obtained after dialysis was 13.82 mg exhibiting a specific 3.56 U/ml of fibrinolytic activity. The protein from each purification step was when subjected for fibrinolytic activity; an increase in halo formation was observed (Table 2). The fibrinolytic activity of the partially purified protein increases with each purification step. In addition, a twofold increment was observed in fibrinolytic activity of an enzyme. Similar increase in the activity was observed in Fu–P, herinase, verticase, and CMase fibrinolytic enzyme (Li et al. 2007; Cui et al. 2008; Wu et al. 2009b; Choi et al. 2013).

**Table 2:** Purification steps of fibrinolytic enzyme from *Lasiodiplodia pseudotheobromae* #1088 AMSTITYEL

Steps	Fibrinolytic activity (in mm^2^)	Total protein (mg)	Activity (U/ml)	Total activity (IU)	Specific activity (U/mg)	Fold	Yield
Culture filtrate	113.04	1804.17	3.196	3196	1.77	1	100
Crude extract	176.62	18.54	5.187	51.87	2.79	1.57	1.62
Dialysed fraction	226.87	13.82	6.514	49.32	3.56	2.01	1.54

The units of activity are calculated on the basis of the plasmin standard

* The activity was determined by the fibrin plate assay as described in “Materials and methods”

**Fig. 3:**
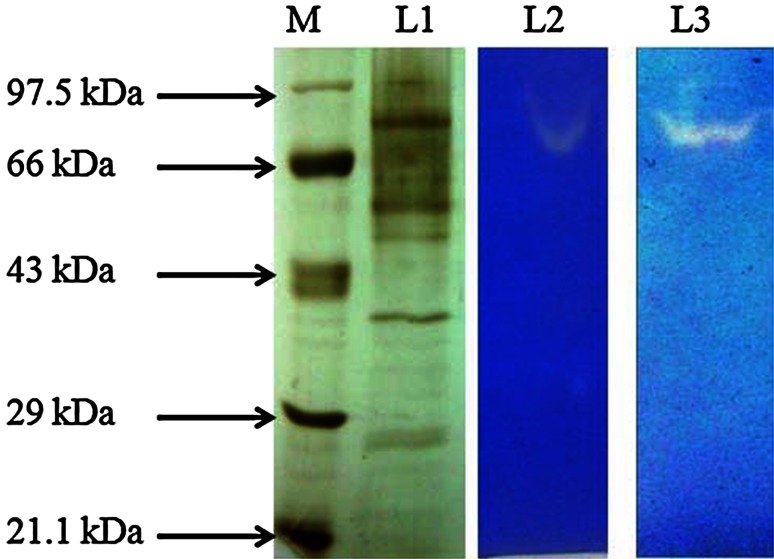
Eletrophorectic analysis of crude enzyme extract of #1088AMSTITYEL. M: Molecular weight marker, Lane L1 resolved protein bands in 10 % SDS-gel, L2: Fibrin hydrolysis exerted by culture filtrate of #1088 AMSTITYEL, L3: Fibrin degradation by crude enzyme extract of #1088 AMSTITYEL in fibrin zymography. White clear areas show fibrin hydrolysis by the active proteins of #1088AMSTITYEL

The crude protein of #1088 AMSTITYEL resolved into six bands of various sizes that ranged from 26  to 80 kDa. According to fibrin zymography, single protein band was responsible for the fibrinolytic activity exhibited by the crude enzyme extract. When the fibrin zymogram was developed, the fibrin degradation was attributable to a protein of molecular weight ~80 KDa (Fig. 3). The molecular weight of the microbial fibrinolytic enzyme varies from 17 to 51 kDa (Peng et al. 2005; Wu et al. 2009b), but the fibrinolytic activity of #1088 AMSTITYEL is attributed to 80-kDa protein, which depicts the novelty of the protein that has yet not been explored.

### Taxonomic identification

Endophytic fungal isolate #1088 AMSTITYEL was identified using molecular and morphotaxonomic tools.

### Molecular taxonomy

**Fig. 4:**
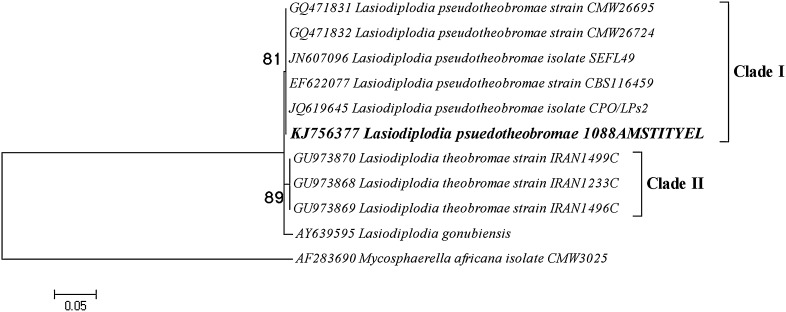
Maximum likelihood tree based on the ITS1–5.8S–ITS2 region of *Lasiodiplodia pseudotheobromae* #1088 AMSTITYEL. The percentage of replicate trees in which the associated taxa clustered together in the bootstrap test (1,000 replicates). 0.05 refers to the distance scale that denotes a 5 % difference between the two sequences

The phylogenetic tree resolved into two major clades: Clade I and Clade II. Clade I clustered #1088 AMSTITYEL along with five other species of *Lasiodiplodia pseudotheobromae*. In Clade II, three species of *L. theobromae* were grouped, and *L. gonubiensis* was resolved basal to the tree. *Mycospherella Africana* was used as outgroup to root the tree (Fig. 4). Thus, the fibrinolytic enzyme producing isolate #1088 AMSTITYEL was identified as *Lasiodiplodia pseudotheobromae*. Other fibrinolytic enzyme producing fungi, such as *Fusarium* sp. BLB and *Fusarium* sp. CPCC 480097, were also identified using similar approach (Ueda et al. 2007; Wu et al. 2009a).

### Morphotaxonomy

**Fig. 5:**
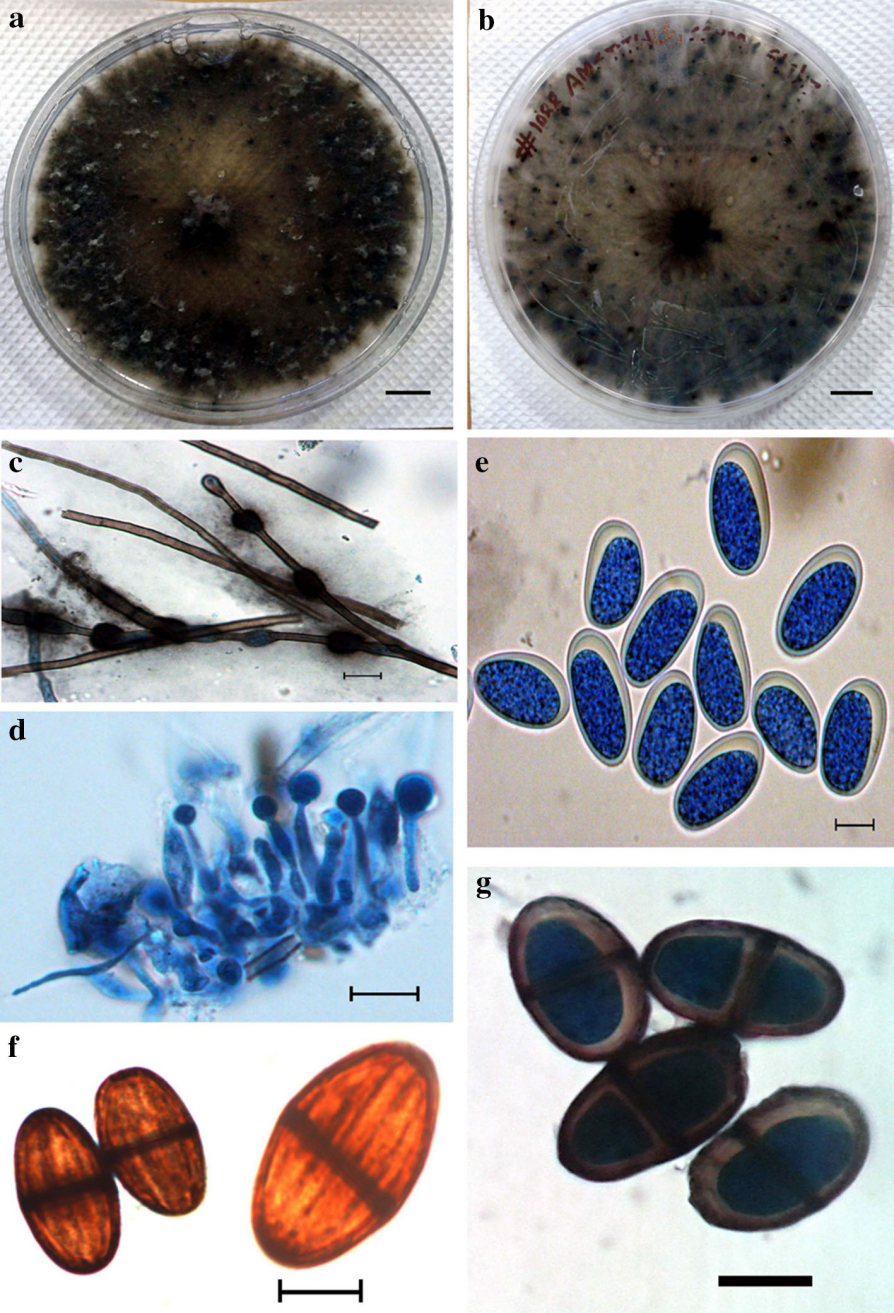
Morphological features of #1088 AMSTITYEL after 30 days of incubation. **a** Colony over PLA plate. **b** Colony reverse view (Bar a–b: 10 mm). **c** Hyphae. **d** Conidiogenous cells with paraphysis. **e** Immature conidia. **f**–**g** Mature conidia (Bar, c–g = 10 µm)

Colonies floccose: grey-to-green coloured, fast growing colony with the formation of aerial hyphae. Hypha (2.66−) 3.83 ± 0.79 (−4.97)-μm thick, highly branched, brown-colored, septate, broad, thick walled. Conidiomata pycnidial, uniloculate, dark brown to black, immersed in the medium becoming erumpent when mature. Paraphyses cylindrical, aseptate, ends rounded up 58-μm long, 3-to-4-μm wide arising from the conidiogenous cells. Conidiogenous cells (11.44–) 17.17 ± 3.14 (–22.04) × (2.89–) 4.98 ± 1.23 (–6.8)-μm hyaline, smooth, cylindrical, slightly swollen at the base, holoblastic, proliferating per currently to form one or two closely spaced annulations. Conidia (23.65–) 27.41 ± 1.7 (–31.69–) × (11.49–) 13.5 ± 0.81 (–14.86) μm, ellipsoidal, apex and base rounded, widest at the middle, thick-walled, initially hyaline and aseptate and remaining so for a long time, becoming one septate and dark brown (24.71–) 28.2 ± 2.18 (–31.07) × (13.5–) 15.87 ± 1.28 (–18.93) μm only some time after release from the conidiomata, with melanin deposits on the inner surface of the wall arranged longitudinally giving a striate appearance to the conidia (Fig. 5). The morphological features of the isolate #1088 AMSTITYEL corresponded with typical *Lasiodiplodia pseudotheobromae* morphology, thereby suggesting it to be a *Lasiodiplodia pseudotheobromae* species.

## Concluding remarks

Thus, this study proves that endophytic fungus *Lasiodiplodia pseudotheobromae* # 1088 AMSTITYEL isolated from *A. marmelos* possesses potential in vitro fibrinolytic potential. Further studies are warranted on purification, characterisation and mass production of bioactive molecules, so that it can be further taken up for preclinical studies.

## Electronic supplementary material

Below is the link to the electronic supplementary material.

Supplementary material 1 (TIFF image 671 kb 368 kb)

Supplementary material 2 (MAS File 8 kb 671 kb 368 kb)

## References

[CR1] Abdollahzadeh J, Javadi A, Goltapeh AM, Zare R, Phillips AJL (2010). Phylogeny and morphology of four new species of *Lasiodiplodia* from Iran. Persoonia.

[CR2] Ali UF, Ibrahim ZM (2008). Production and some properties of fibrinolytic enzyme from *Rhizomucor miehei* (Cooney & Emerson) Schipper. J Appl Sci Res.

[CR3] Astrup T, Mullertz S (1952). The fibrin plate method for estimating fibrinolytic activity. Arch Biochem Biophys.

[CR4] Blum H, Beier H, Gross HJ (1987). Improved silver staining of plant proteins, RNA and DNA in polyacrylamide gels. Electrophoresis.

[CR5] Bradford MM (1976). A rapid and sensitive method for the quantification of microgram quantities of proteins utilizing the principle of protein-dye binding. Anal Biochem.

[CR6] Choi DB, Cha WS, Park N, Kim HW, Lee JH, Park JS, Park SS (2011). Purification and characterization of a novel fibrinolytic enzyme from fruiting bodies of Korean *Cordyceps militaris*. Bioresour Technol.

[CR7] Choi BS, Sapkota K, Choi JH, Shin C, Kim S, Kim SJ (2013). Herinase: a Novel Bi-functional Fibrinolytic Protease from the Monkey Head Mushroom, *Hericium erinaceum*. Appl Biochem Biotechnol.

[CR8] Chougule P, Jain V, Suryawanshi A, Jain A (2014). Screening of thrombolytic activity of Aegle marmelos Linn leaves extract in vitro assay. Int J Pharm Sci Rev Res.

[CR9] Cui L, Dong MS, Chen XH, Jiang M, Lv X, Yan G (2008). A novel fibrinolytic enzyme from *Cordyceps militaris*, a Chinese traditional mushroom. World J Microbiol Biotechnol.

[CR32] Daisy B, Strobel G (2003). Bioprospecting for microbial endophytes and their natural products. Microbiol Mol Biol Rev.

[CR10] Deng Z, Wang S, Li Q, Ji X, Zhang L, Hong M (2010). Purification and characterization of a novel fibrinolytic enzyme from the polychaete, *Neanthes japonica* (Iznka). Bioresour Technol.

[CR11] Gond SK, Verma VC, Kumar A, Kumar V, Kharwar RN (2007). Study of endophytic fungal community from different parts of *Aegle marmelos* Correa (Rutaceae) from Varanasi (India). World J Microb Biol.

[CR12] Kim W, Choi K, Kim Y, Park H, Choi J, Lee Y (1996). Purification and characterization of a fibrinolytic enzyme produced from *Bacillus* sp. strain CK 11-4 screened from Chungkook-Jang. Appl Environ Microbiol.

[CR13] Kim SH, Choi NS, Lee WY (1998). Fibrin zymogyaphy: a direct analysis of fibrinolytic enzymes on gels. Anal Biochem.

[CR14] Laemmli UK (1970). Cleavage of structural proteins during the assembly of the head of bacteriophage T4. Nature (Lond).

[CR15] Li Y, Shuang JL, Yuan WW, Huang WY, Tan RX (2007). Verticase: a fibrinolytic enzyme produced by *Verticillium* sp. Tj33, an endophyte of *Trachelospermum jasminoides*. J Integr Plant Biol.

[CR16] Mander P, Cho SS, Simkhada JR, Choi YH, Yoo JC (2011). A low molecular weight chymotrypsin-like novel fibrinolytic enzyme from *Streptomyces* sp. CS624. Process Biochem.

[CR17] Meshram V, Sanjai Saxena, Kapoor N (2012). In *vitro* anti-staphylococcal potential of endophytic fungi from *Aegle marmelos*. J Pure App Microbiol.

[CR18] Naik BS, Shashikala J, Krishnamurthy YL (2008). Diversity of fungal endophytes in shrubby medicinal plants of Malnad region, Western Ghats, Southern India. Fungal ecol.

[CR19] Nei N, Kumar S (2000). Molecular Evolution and Phylogenetics.

[CR20] Peng Y, Yang X, Zhang Y (2005). Microbial fibrinolytic enzymes: an overview of source, production, properties, and thrombolytic activity *in vivo*. Appl Microbiol Biotechnol.

[CR21] Raafat AI, Araby E, Lotfy S (2012). Enhancement of fibrinolytic enzyme production from *Bacillus subtilis* via immobilization process onto radiation synthesized starch/dimethylaminoethyl methacrylate hydrogel. Carbohyd Polym.

[CR22] Rovati JI, Delgado OD, Figueroa LIC, Farina JI (2010). A novel source of fibrinolytic activity: *bionectria* sp., an unconventional enzyme-producing fungus isolated from Las Yungas rainforest (Tucuma´n, Argentina). World J Microbiol Biotechnol.

[CR23] Schulz B, Wanke U, Draeger S, Aust HJ (1993). Endophytes from herbaceous plants and shrubs: effectiveness of surface sterilization methods. Myco Research.

[CR24] Simkhada JR, Mander P, Cho SS, Yoo JC (2010). A novel fibrinolytic protease from *Streptomyces* sp. CS684. Process Biochem.

[CR25] Tamura K, Peterson D, Peterson N, Stecher G, Nei M, Kumar S (2011). MEGA5: molecular Evolutionary Genetics Analysis using Maximum Likelihood, Evolutionary Distance, and Maximum Parsimony Methods. Mol Biol Evol.

[CR26] Ueda M, Kubo T, Miyatake K, Nakamura T (2007). Purification and characterization of fibrinolytic alkaline protease from *Fusarium* sp. BLB. Appl Microbiol Biotechnol.

[CR27] White TJ, Bruns T, Lee S, White BA (1990). Taylor J (1990) Amplification and direct sequencing of fungal ribosomal RNA genes for phylogenetics. PCR protocols: a guide to methods and applications.

[CR28] Wu B, Wu L, Ruan L, Gei M, Chen D (2009). Screening of endophytic fungi with antithrombotic activity and identification of a bioactive metabolite from the endophytic fungal strain CPCC 480097. Curr Microbiol.

[CR29] Wu B, Wu L, Chen D, Yang Z, Luo M (2009). Purification and characterization of a novel fibrinolytic protease from *Fusarium* sp. CPCC 480097. J Ind Microbiol Biotechnol.

[CR30] Zhang HW, Song YC, Tan RX (2006). Biology and chemistry of endophytes. Nat Prod Rep.

[CR31] Zhang CL, Wang GP, Mao LJ, Komon- Zelazowska M, Yuan ZL, Lin FC, Druzhinina IS, Kubicek CP (2010). *Muscodor fengyangensis* sp. nov. from south east China: morphology, physiology and production of volatile compounds. Fungal Biol.

