# Erratum to: Potential fibrinolytic activity of an endophytic *Lasiodiplodia pseudotheobromae* species

**DOI:** 10.1007/s13205-016-0451-5

**Published:** 2016-06-14

**Authors:** Vineet Meshram, Sanjai Saxena

**Affiliations:** Department of Biotechnology, Thapar University, Patiala, Punjab 147004 India

## Erratum to: 3 Biotech (2016) 6:114 DOI 10.1007/s13205-016-0428-4

Unfortunately, the Fig. 2 legend was published incorrectly in the original version of the article. The correct Fig. 2 legend is given below:

**Fig. 2** Screening culture filtrates of endophytic fungal isolates of *A. marmelos* for fibrinolytic activity. Well id: *a*–*c* #1088 AMSTITYEL; *d*–*f* #1048 AMSTITYEL, *g*–*i* #1095 AMSTITWLS; *j*–*l* #1013 AMSTITYEL, m: control

