# Supplementary figures and images for: Potential fibrinolytic activity of an endophytic Lasiodiplodia pseudotheobromae species
Source: 3 Biotech. 2016 May 19;6(1):114. doi: 10.1007/s13205-016-0428-4 (PMC5398192; doi:10.1007/s13205-016-0428-4)

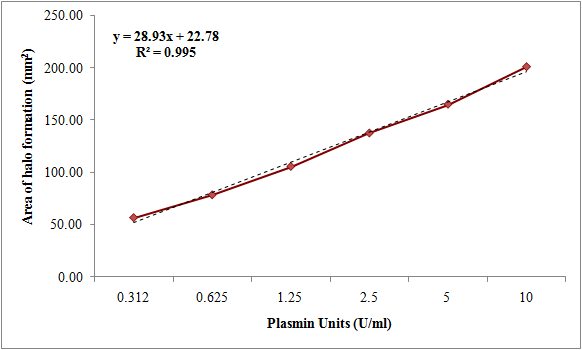

Supplement: Supplementary file 1 — Supplementary material 1 (TIFF image 671 kb 368 kb) [file 13205_2016_428_MOESM1_ESM.tif]
